# Associations between weight-adjusted-waist index and infertility: Results from NHANES 2013 to 2020

**DOI:** 10.1097/MD.0000000000036388

**Published:** 2023-12-01

**Authors:** Huanxin Zhong, Bin Yu, Fen Zhao, Hongyin Cui, Lifang You, Dao Feng, Yi Lu

**Affiliations:** a Gynecology Department, Linping Campus, Second Affiliated Hospital, Zhejiang University School of Medicine, Hangzhou, China; b Thyroid & Breast Surgery, Linping Campus, Second Affiliated Hospital, Zhejiang University School of Medicine, Hangzhou, China.

**Keywords:** cross-section study, infertility, NHANES, obesity, weight-adjusted waist circumference

## Abstract

Female infertility is a significant problem for women of reproductive age worldwide. Obesity has been proven to pose a danger for infertility in women. Weight-adjusted waist circumference index (WWI) is a recently created biomarker of obesity, and this research aims to explore the relationship between female infertility and WWI. Data for this investigation were gathered from National Health and Nutrition Examination Survey. We used weighted multivariate logistic regression, subgroup analysis, interaction testing, and smoothed curve fitting to investigate the relationship between infertility and WWI. A total of 6333 women were included and 708 (11.18%) had infertility. It was discovered that women with higher WWI had increased probabilities of infertility (OR = 1.92, 95% CI: 1.42–2.59) adjusting for confounders. In addition, WWI was linked to increased chances of infertility in women aged 28 to 36 years (OR = 1.59, 95% CI: 1.28–1.97). According to the results of this cross-sectional survey, WWI is positively associated with infertility among adult females in the U.S. And it can help identify infertile women and may help reduce the risk of infertility.

## 1. Introduction

Infertility is the inability to become pregnant after a year of regular, unprotected sexual activity.^[1]^ An identifiable reason can be found in 85% of infertile couples. The remaining 15% of infertile couples experience “unexplained infertility.” Smoking and obesity are 2 lifestyle and environmental variables that might harm fertility.^[2,3]^ Infertility has become a global public health issue. The physical and mental health of a woman is affected, and infertility has substantial public health repercussions.^[4]^

A worldwide epidemic of obesity has emerged. According to the World Health Organization, 60% of women in Europe and the U.S are obese. In comparison, obesity now affects more than 20% of American women of childbearing age and affects 30% of women overall.^[5,6]^ The adverse effects of obesity on reproductive physiology are known, as obese women often experience irregular menstruation, impaired ovulation, and endometriosis.^[7,8]^ In spite of its popularity as a measure of obesity,^[9]^ body mass index (BMI) is limited in its ability to distinguish between lean and fat tissues.^[10–12]^ Waist circumference (WC) as a predictor of obesity is associated with visceral fat and abdominal obesity.^[13]^ In 2018, Park et al introduced Weight-adjusted waist circumference index (WWI) (cm/√kg), a novel measure for measuring obesity based on WC divided by the square root of body weight,^[12]^ which reflects both the components of muscle and fat mass, irrespective of BMI category.^[14,15]^ A previous study showed that a positive association between WWI and fat mass, while an inverse association was observed with muscle mass.^[16]^ Thus, WWI indicates central obesity, however, no research has ever investigated the connection between infertility and WWI. In contrast, WWI may be an essential tool for assessing health risks.^[17]^

To increase understanding of obesity negative impact on infertility, it is essential to explore the relationship between infertility and WWI. Therefore, we investigate the relationship between WWI and infertility using the 2013 to 2020 National Health and Nutrition Examination Survey (NHANES), and we suppose that WWI levels would be positively correlated with the prevalence of infertility.

## 2. Methods

### 2.1. Study participants

The studied data were taken from NHANES database, a significant nationwide initiative run by the National Center for Health Statistics of the Centers for Disease Control and Prevention to evaluate the level of nutrition and health in the American population. The survey unique aspect is its seamless blending of physical checks and interviews. Concerns about demographics, socioeconomics, diet, and health are covered in the survey interview. In addition, the examination components also include laboratory analysis conducted by highly qualified medical professionals, along with medical, dental, and physiological evaluations. All participants in the NHANES study protocols that were authorized by the Research Ethics Review Board for National Center for Health Statistics signed the informed consent form. The public can access more in-depth information about the NHANES survey at https://www.cdc.gov/nchs/nhanes/index.htm.

**Figure 1.**
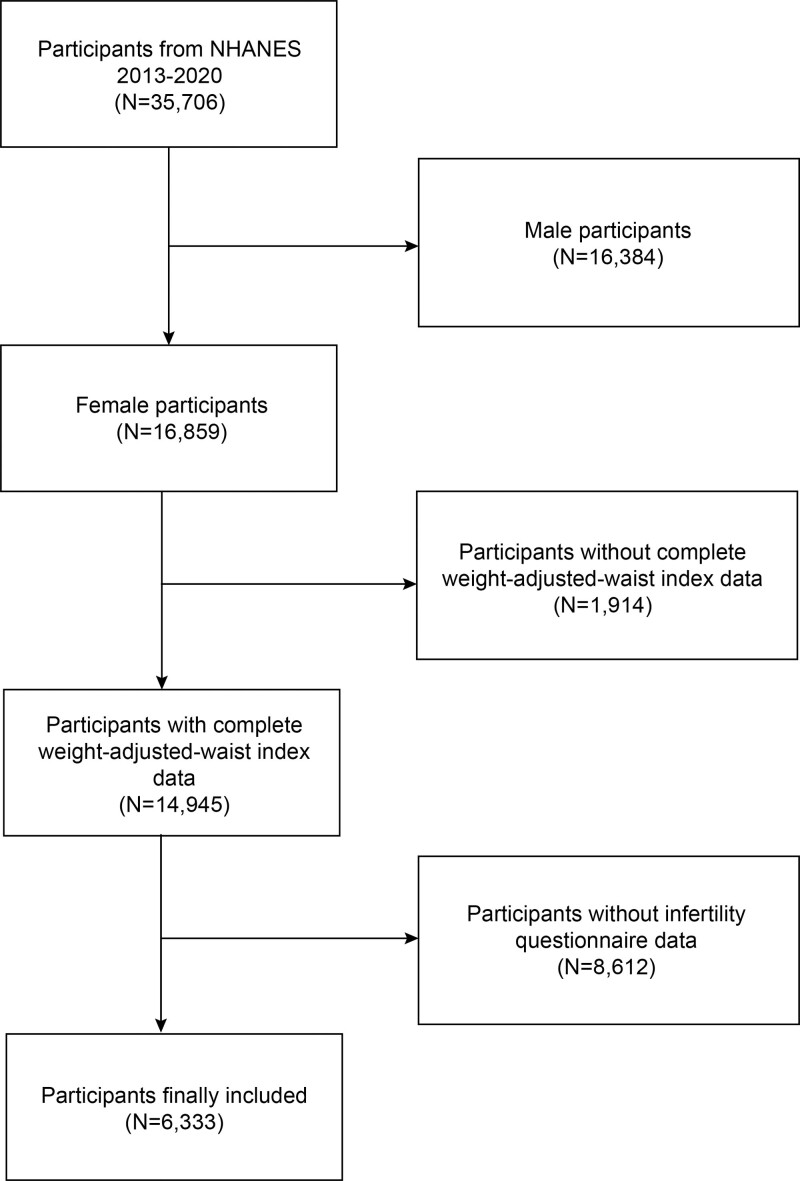
Flow chart of participants selection. NHANES = National Health and Nutrition Examination Survey.

The data from 4 survey cycles (2013–2020) were used in this cross-sectional analysis. A total of 35,706 research participants were screened. We excluded men (n = 16,384), missing WWI data (n = 1914), and without completing the infertility questionnaire (n = 8612) were excluded. In the end, 6333 people were included in our research (Fig. 1).

### 2.2. Weight-adjusted waist circumference index

WWI which gauges obesity by normalizing the WC of body weight, is computed as the square root of WC (cm) divided by body weight (kg). Health technicians in the MEC with the appropriate training measured participants’ weight and WC. For analysis, the participants were categorized into 4 groups (Q1–Q4) based on the quartiles of the WWI. In our research, WWI was considered as an exposure and was thought to be a continuous variable.

### 2.3. Infertility

Infertility was the study outcome variable (questionnaire variable name: RHQ074). Infertility was assumed to exist in those who responded positively to the survey question “Have you ever tried to become pregnant for at least a year without becoming pregnant?.”^[18]^

### 2.4. Covariables

Covariates included age, race, BMI, family income-to-poverty ratio, Triglycerides, low-density lipoprotein cholesterol, Alcohol intaking, Education level, Smoking, Diabetes, and Marital Status.

### 2.5. Statistical analysis

EmpowerStats (version 5.0) and R (version 4.2) software were used to conduct the statistical analysis. To evaluate participant demographic characteristics by WWI quartiles, we performed chi-square tests and *t* tests. We investigated the linear link between WWI and infertility using weighted multiple logistic regression analysis. To investigate trends in the linear relationship between female infertility and WWI, we used a trend test after dividing WWI into quartiles. In addition, we conducted subgroup analyses based on age, race, marital status, education, and diabetes status to examine the relationship between female infertility and WWI in different subpopulations. To evaluate the consistency of the association between the other groupings, we also performed interaction tests. We also used smoothed curve fitting to investigate the nonlinear relationship between female infertility and WWI. A 2-sided *P* < .05 was utilized as statistical significance.^[19,20]^

## 3. Results

### 3.1. Baseline characteristics

Based on the inclusion and exclusion criteria, 6333 individuals were included in the study, with 708 infertility patients (11.8%) and a mean (SD) age of 38.29 ± 11.69 years. Mexican Americans (15.68%), non-Hispanic Blacks (24.04%), non-Hispanic Whites (32.81%), and Other Races (27.47%). The mean (SD) WWI for all individuals is 11.01 (0.85).

The clinical features of the study subjects are presented in Table 1, and the classification based on WWI stratified the participants into 4 groups of equal size. Participants in the highest WWI quartile had a higher likelihood of Mexican Americans and older than those in the lowest quartile. Participants with a high level of WWI had lower levels of income and education. Along with having a higher BMI, triglycerides, and low-density lipoprotein cholesterol, they also had a higher frequency of diabetes and a higher chance of infertility.

**Table 1:** Basic characteristics of participants by weight-adjusted-waist index quartile.

Characteristics	Weight-adjusted-waist index				*P* value
	Q1 (<10.45) N = 1583	Q2 (10.45–11.03) N = 1583	Q3 (11.04–11.62) N = 1583	Q4 (>11.62) N = 1584	
Age (yr)	32.02 ± 11.48	37.74 ± 11.97	40.51 ± 11.79	42.90 ± 11.53	<0.001
BMI (kg/m^2^)	24.44 ± 5.68	28.01 ± 6.55	31.13 ± 7.29	36.32 ± 8.53	<0.001
Family PIR	2.59 ± 1.64	2.55 ± 1.60	2.35 ± 1.54	2.08 ± 1.47	<0.001
Triglycerides (mg/dL)	73.39 ± 26.83	83.78 ± 40.51	93.99 ± 113.53	101.21 ± 59.72	<0.001
LDL-C (mg/dL)	101.69 ± 19.73	107.24 ± 22.93	109.49 ± 22.53	110.66 ± 23.51	<0.001
Alcohol intaking, (%)	3.02 ± 28.52	3.12 ± 29.44	4.98 ± 52.01	2.42 ± 1.88	0.314
Race/ethnicity, (%)					<0.001
Non-Hispanic White	588 (37.14%)	527 (33.29%)	445 (28.11%)	518 (32.70%)	
Non-Hispanic Black	428 (27.04%)	368 (23.25%)	368 (23.25%)	358 (22.60%)	
Mexican American	130 (8.21%)	195 (12.32%)	327 (20.66%)	341 (21.53%)	
Other race/multiracial	437 (27.61%)	493 (31.14%)	443 (27.98%)	367 (23.17%)	
Education level, n (%)					<0.001
Less than high school	116 (8.66%)	172 (11.60%)	305 (20.12%)	363 (23.42%)	
High school	222 (16.58%)	301 (20.30%)	309 (20.38%)	376 (24.26%)	
More than high school	1001 (74.76%)	1010 (68.11%)	902 (59.50%)	811 (52.32%)	
Smoking, (%)					<0.001
Ever	1208 (76.31%)	1120 (70.80%)	1068 (67.51%)	1011 (63.83%)	
Never	375 (23.69%)	462 (29.20%)	514 (32.49%)	573 (36.17%)	
Diabetes, (%)					<0.001
Yes	25 (1.58%)	86 (5.43%)	177 (11.20%)	322 (20.37%)	
Borderline					
No	1558 (98.42%)	1497 (94.57%)	1403 (88.80%)	1259 (79.63%)	
Marital status, (%)					<0.001
Married/living with partner	702 (52.43%)	873 (58.87%)	925 (61.02%)	902 (58.19%)	
Widowed/divorced/separated	381 (28.45%)	401 (27.04%)	441 (29.09%)	473 (30.52%)	
Never married	256 (19.12%)	209 (14.09%)	150 (9.89%)	175 (11.29%)	
Infertility, (%)					<0.001
Yes	121 (7.64%)	176 (11.12%)	195 (12.32%)	216 (13.64%)	
No	1462 (92.36%)	1407 (88.88%)	1388 (87.68%)	1368 (86.36%)	

Mean ± SD for continuous variables: the *P* value was calculated by the weighted linear regression model. (%) for categorical variables: the *P* value was calculated by the weighted chi-square test.

BMI = body mass index, LDL-C = low-density lipoprotein cholesterol, PIR = Ratio of family income to poverty, Q = quartile.

### 3.2. The associations between WWI and infertility.

Table 2 displays the outcomes of the multiple regression analysis. In the model without adjustment [1.30 (1.19, 1.43)], WWI is positively associated with infertility. Additionally, even after controlling for the corresponding factors in Models 2 [1.25 (1.13, 1.38)] and Model 3 [1.33 (1.17, 1.50)], this connection remained significant. In the completely revised model (Model 3), each unit increase in WWI was related to a 33% greater risk of infertility [1.33 (1.17, 1.50)]. In model 3, those in the highest quartile of the survey (Q4) had a 92% greater probability of experiencing infertility than those in the lowest quartile (Q1) (OR = 1,92, 95% CI:1.42, 2.59).

**Table 2:** The associations between weight-adjusted-waist index and infertility.

Exposure	Model 1 [OR (95% CI)]	Model 2 [OR (95% CI)]	Model 3 [OR (95% CI)]
WWI (continuous)	1.30 (1.19, 1.43)	1.25 (1.13, 1.38)	1.33 (1.17, 1.50)
WWI (quartile)			
Quartile 1	Reference	Reference	Reference
Quartile 2	1.51 (1.19, 1.93)	1.44 (1.12, 1.84)	1.40 (1.06, 1.87)
Quartile 3	1.70 (1.34, 2.15)	1.61 (1.25, 2.06)	1.69 (1.26, 2.25)
Quartile 4	1.91 (1.51, 2.41)	1.73 (1.35, 2.21)	1.92 (1.42, 2.59)
*P* for trend	<.001	<.001	<.001

Model 1: no covariates were adjusted. Model 2: age and race were adjusted. Model 3: age, race, BMI, Family PIR, Triglycerides, LDL-C, Alcohol intaking, Education level, Smoking, Diabetes, Marital Status.

WWI = weight-adjusted waist circumference index.

### 3.3. Table 3 subgroup analysis

We performed subgroup analyses to determine whether the WWI-infertility association was constant across strata. WWI indicated a substantial positive connection with infertility in all subgroups. Table 3 shows that no subgroup, including race, education, marital status, or diabetes, had an impact on the independently positive connection between infertility and WWI (all *P* > .05 for interaction).

**Table 3:** Subgroup analysis of the association between weight-adjusted-waist index and infertility.

Subgroup	Infertility [OR (95%CI)]	*P* for interaction
Age		.013
≤27 yr	1.26 (0.93, 1.70)	
28–36 yr	1.59 (1.28, 1.97)	
>36 yr	1.08 (0.94, 1.24)	
Race/ethnicity		.996
Non-Hispanic White	1.26 (1.06, 1.49)	
Non-Hispanic Black	1.24 (1.01, 1.53)	
Mexican American	1.20 (0.88, 1.65)	
Other race	1.26 (1.00, 1.58)	
Education level		.146
Less than high school	1.51 (1.10, 2.07)	
High school	1.04 (0.82, 1.32)	
More than high school	1.28 (1.13, 1.46)	
Marital status		.198
Married/living with partner	1.23 (1.07, 1.40)	
Widowed/divorced/separated	1.41 (1.14, 1.75)	
Never married	0.96 (0.66, 1.40)	
Diabetes		.235
Yes	1.03 (0.74, 1.44)	
No	1.27 (1.14, 1.43)	

Race, age, BMI, smoking, Education level, Marital Status, PIR, LDL-C, and triglycerides were adjusted.

**Figure 2.**
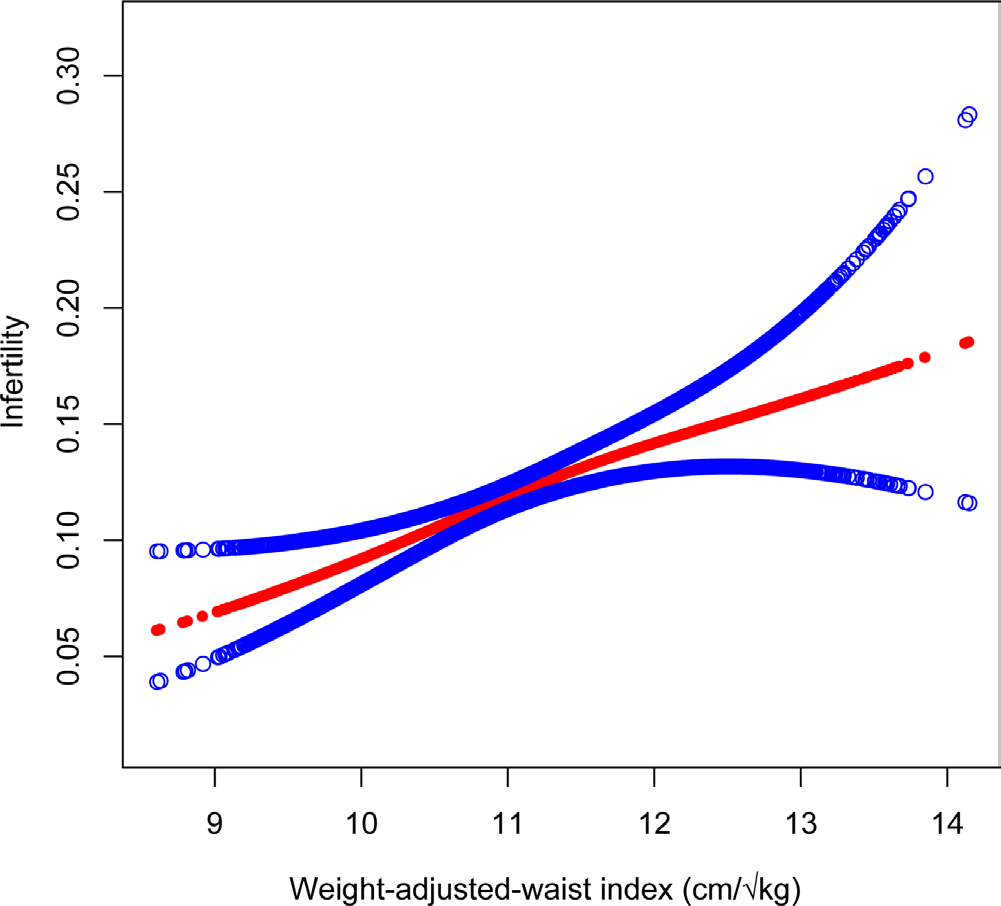
Non-linear relationship between weight-adjusted waist circumference index and infertility. The solid red line represents a smooth curve fit between the variables. The blue bars represent the 95% confidence intervals of the fitted results. WWI = weight-adjusted waist circumference index.

The nonlinear positive connection between infertility and WWI has been confirmed at all 4 sites by smoothed curve fitting results (Fig. 2).

## 4. Discussion

In this cross-sectional study, which included 6333 participants, we explored the association between infertility and WWI in US adults and found a favorable correlation between infertility and WWI. Subgroup analysis showed that all stratification variables had no impact on the stability of the connection between WWI and infertility and the positive association persisted even after WWI was converted into a categorical variable by quartiles (Q1–Q4). Additionally, we discovered proof of a substantial link between infertility and WWI in participants between 28 and 36 years. Finally, we used a smoothing curve to further establish the nonlinear relationship between female infertility and WWI.

As far as we know, this is the first research to explore the association between female infertility and WWI, and it emphasizes the positive association of WWI levels with a higher risk of infertility. Previous research has shown that obesity negatively impacts infertility.^[21,22]^ According to a large U.S. cohort of more than 7000 women, menstruating obese women had lower fertility, according to Gesink Law et al In a large cohort study from the Netherlands by Van der Steeg et al’s, a cohort of more than 3000 women with regular menstrual cycles showed that every BMI above 29 kg/m^2^ linearly decreased the likelihood of spontaneous conception.^[23,24]^ After adjustment for BMI, another US prospective cohort found attenuated negative correlations between WC and waist-to-hip ratio and fertility.^[25]^ These results imply that the association between BMI and females” infertility is more complex than a simple linear positive correlation can capture. Numerous research has discovered saturation effects and nonlinear correlations between BMI and infertility among people of different ages, genders and various ethnic groups.^[19,26]^

Currently, most studies use BMI and abdominal circumference to assess the degree of obesity; however, obese patients, due to lack of physical activity, tend to exhibit fat accumulation, which further raises the possibility of infertility development in non-obese patients.^[18,20]^ Additionally, several research have investigated the connection between fertility rates and central obesity, specifically deep subcutaneous tissue fat buildup, which has been suggested as a possible trigger for the onset of infertility.^[27–29]^ The status of obese people may thus not be fully reflected by BMI computed using total weight. While central obesity is largely represented by WC, which is computed by normalizing it to body weight, WWI allows for the assessment of high-fat mass and low muscle mass,^[14]^ which has been investigated in several fields and predicts total body fat percentage more accurately.^[30,31]^ For instance, Ding et al reported no association between BMI and WC and a nonlinear positive association between WWI levels and the risk of cardiovascular disease and all-cause mortality.^[30]^ Additionally, Qin et al found a higher correlation with the prevalence of proteinuria than additional fat-related indicators. This shows that WWI, as opposed to other indicators of fat, such BMI, is a stronger predictor of albuminuria.^[31]^

Infertility is widespread, and the underlying causes of this unfavorable relationship between females’ infertility and WWI are uncertain. Body fat in the abdomen differs metabolically from subcutaneous fat, and central obesity is characterized by the excessive buildup of adipose tissue in the abdominal region, particularly in the visceral area. Visceral adipose tissue exhibits a consistent ability to secrete pro-inflammatory stimulants, which subsequently accumulate in various tissues and give rise to detrimental effects known as lipotoxicity.^[32]^ Adipotoxicity plays a role in the increased systemic inflammatory response and the development of insulin resistance in obese women as a potential mechanism for obesity-induced organelle damage in oocytes.^[33]^ Other studies have shown that the predisposition to infertility in obese patients may be associated with functional changes in the hypothalamic-pituitary-ovarian axis. Tortoriello et al designed an experimental animal study to show that obese (diet-induced obesity) mice have a 60% lower pregnancy rate when induced by diet, however, this defect can be overcome using exogenous gonadotropins.^[34,35]^

This study has several limitations that need to be mentioned. First, since the study was cross-sectional, a causal relationship between WWI and female infertility could not be established. Second, we should proceed with caution because several infertility-related factors, like menarche and medication use, could not be studied due to database constraints. There are many advantages to our research despite these limitations. The 2 strengths of our study are the use of a sophisticated multi-stage random sampling strategy and a sufficiently large sample size. After adjusting for confounders, we used subgroup analyses to determine the reliability of the regression analyses.

## 5. Conclusion

In conclusion, our study found that elevated levels of WWI were strongly associated with the risk of developing infertility, as we found a striking positive correlation between infertility and WWI among U.S. adults. It might eventually be utilized as a straightforward anthropometric indicator to forecast infertility.

## Acknowledgments

I would like to thank all participants in this study.

## Author contributions

**Data curation:** Huanxin Zhong.

**Formal analysis:** Huanxin Zhong, Hongyin Cui, Yi Lu.

**Funding acquisition:** Yi Lu.

**Investigation:** Hongyin Cui, Lifang You, Dao Feng, Yi Lu.

**Methodology:** Bin Yu, Lifang You, Dao Feng.

**Project administration:** Bin Yu, Lifang You, Dao Feng.

**Resources:** Fen Zhao.

**Software:** Bin Yu, Fen Zhao.

**Supervision:** Bin Yu, Fen Zhao.

**Validation:** Fen Zhao.

## References

[1] Ethics Committee of American Society for Reproductive Medicine. Access to fertility treatment by gays, lesbians, and unmarried persons: a committee opinion. Fertil Steril. 2013;100:1524–7. 24094420 10.1016/j.fertnstert.2013.08.042

[2] Carson SA Kallen AN. Diagnosis and management of infertility: a review. JAMA. 2021;326:65–76. 34228062 10.1001/jama.2021.4788 PMC9302705

[3] Bellver J Donnez J. Introduction: infertility etiology and offspring health. Fertil Steril. 2019;111:1033–5. 31155112 10.1016/j.fertnstert.2019.04.043

[4] Inhorn MC Patrizio P. Infertility around the globe: new thinking on gender, reproductive technologies and global movements in the 21st century. Hum Reprod Update. 2015;21:411–26. 25801630 10.1093/humupd/dmv016

[5] World Health Organization. Obesity and overweight fact sheet 2016. Available at: http://www.who.int/mediacentre/factsheets/fs311/en/. [access date Mar 6, 2017].

[6] Knight M Kurinczuk JJ Spark P. Extreme obesity in pregnancy in the United Kingdom. Obstet Gynecol. 2010;115:989–97. 20410773 10.1097/AOG.0b013e3181da8f09

[7] Greil AL Johnson KM McQuillan J. Are prior pregnancy outcomes relevant for models of fertility-specific distress or infertility helpseeking? Hum Fertil (Camb). 2011;14:160–6. 21732891 10.3109/14647273.2011.587229

[8] Talmor A Dunphy B. Female obesity and infertility. Best Pract Res Clin Obstet Gynaecol. 2015;29:498–506. 25619586 10.1016/j.bpobgyn.2014.10.014

[9] De Lorenzo A Gratteri S Gualtieri P. Why primary obesity is a disease? J Transl Med. 2019;17:169. 31118060 10.1186/s12967-019-1919-y PMC6530037

[10] Oliveros E Somers VK Sochor O. The concept of normal weight obesity. Prog Cardiovasc Dis. 2014;56:42633. 10.1016/j.pcad.2013.10.003 24438734

[11] Cai S Zhou L Zhang Y. Association of the weight-adjusted-waist index with risk of all-cause mortality: a 10-year follow-up study. Front Nutr. 2022;9:894686. 35694172 10.3389/fnut.2022.894686 PMC9174751

[12] Park Y Kim NH Kwon TY. A novel adiposity index as an integrated predictor of cardiometabolic disease morbidity and mortality. Sci Rep. 2018;8:16753. 30425288 10.1038/s41598-018-35073-4 PMC6233180

[13] Ness-Abramof R Apovian CM. Waist circumference measurement in clinical practice. Nutr Clin Pract. 2008;23:397–404. 18682591 10.1177/0884533608321700

[14] Kim NH Park Y Kim NH. Weight-adjusted waist index reflects fat and muscle mass in the opposite direction in older adults. Age Ageing. 2021;50:780–6. 33035293 10.1093/ageing/afaa208

[15] Kim JY Choi J Vella CA. Associations between weight-adjusted waist index and abdominal fat and muscle mass: multi-ethnic study of atherosclerosis. Diabetes Metab J. 2022;46. 10.4093/dmj.2021.0294 PMC9532169 35350091

[16] Kim JY Choi J Vella CA. Associations between weight-adjusted waist index and abdominal fat and muscle mass: multi-ethnic study of atherosclerosis. Diabetes Metab J 2022;46:747–55. 35350091 10.4093/dmj.2021.0294 PMC9532169

[17] Li Q Qie R Qin P. Association of weight-adjusted-waist index with incident hypertension: the Rural Chinese Cohort Study. Nutr Metab Cardiovasc Dis. 2020;30:1732–41. 32624344 10.1016/j.numecd.2020.05.033

[18] Moran LJ Norman RJ Teede HJ. Metabolic risk in PCOS: phenotype and adiposity impact. Trends Endocrinol Metab. 2015;26:136–43. 25591984 10.1016/j.tem.2014.12.003

[19] Loy SL Cheung YB Soh SE. Female adiposity and time-to-pregnancy: a multiethnic prospective cohort. Hum Reprod. 2018;33:2141–9. 30285230 10.1093/humrep/dey300 PMC6201836

[20] Jungheim ES Moley KH. Current knowledge of obesity’s effects in the pre and periconceptional periods and avenues for future research. Am J Obstet Gynecol. 2010;203:525–30. 20739012 10.1016/j.ajog.2010.06.043 PMC3718032

[21] Broughton DE Moley KH. Obesity and female infertility: potential mediators of obesity’s impact. Fertil Steril. 2017;107:840–7. 28292619 10.1016/j.fertnstert.2017.01.017

[22] Grindler NM Moley KH. Maternal obesity, infertility and mitochondrial dysfunction: potential mechanisms emerging from mouse model systems. Mol Hum Reprod. 2013;19:486–94. 23612738 10.1093/molehr/gat026 PMC3712655

[23] Wise LA Rothman KJ Mikkelsen EM. An internet-based prospective study of body size and time-to-pregnancy. Hum Reprod. 2010;25:253–64. 19828554 10.1093/humrep/dep360 PMC2794667

[24] Ramlau-Hansen CH Thulstrup AM Nohr EA. Subfecundity in overweight and obese couples. Hum Reprod. 2007;22:1634–7. 17344224 10.1093/humrep/dem035

[25] McKinnon CJ Hatch EE Rothman KJ. Body mass index, physical activity and fecundability in a North American preconception cohort study. Fertil Steril. 2016;106:451–9. 27125230 10.1016/j.fertnstert.2016.04.011

[26] Wise LA Palmer JR Rosenberg L. Body size and time-to-pregnancy in black women. Hum Reprod. 2013;28:2856–64. 23958939 10.1093/humrep/det333 PMC3777573

[27] Alvarez-Blasco F Botella-Carretero JI San Millan JL. Prevalence and characteristics of the polycystic ovary syndrome in overweight and obese women. Arch Intern Med. 2006;166:2081–6. 17060537 10.1001/archinte.166.19.2081

[28] van der Steeg JW Steures P Eijkemans MJ. Obesity affects spontaneous pregnancy chances in subfertile, ovulatory women. Hum Reprod. 2008;23:324–8. 18077317 10.1093/humrep/dem371

[29] Chavarro JE Ehrlich S Colaci DS. Body mass index and short-term weight change in relation to treatment outcomes in women undergoing assisted reproduction. Fertil Steril. 2012;98:109–16. 22607889 10.1016/j.fertnstert.2012.04.012 PMC3389169

[30] Ding C Shi Y Li J. Association of weight-adjusted-waist index with all-cause and cardiovascular mortality in China: a prospective cohort study. Nutr Metab Cardiovasc Dis. 2022;32:1210–7. 35277327 10.1016/j.numecd.2022.01.033

[31] Qin Z Chang K Yang Q. The association between weight-adjusted-waist index and increased urinary albumin excretion in adults: a population-based study. Front Nutr. 2022;9:941926. 36034904 10.3389/fnut.2022.941926 PMC9412203

[32] Sorensen TI Virtue S Vidal-Puig A. Obesity as a clinical and public health problem: is there a need for a new definition based on lipotoxicity effects? Biochim Biophys Acta. 2010;1801:400–4. 20045743 10.1016/j.bbalip.2009.12.011

[33] Virtue S Vidal-Puig A. Adipose tissue expandability, lipotoxicity and the metabolic syndrome—an allostatic perspective. Biochim Biophys Acta. 2010;1801:338–49. 20056169 10.1016/j.bbalip.2009.12.006

[34] Tortoriello DV McMinn J Chua SC. Dietary-induced obesity and hypothalamic infertility in female DBA/2J mice. Endocrinology. 2004;145:1238–47. 14670988 10.1210/en.2003-1406

[35] Tortoriello DV McMinn JE Chua SC. Increased expression of hypothalamic leptin receptor and adiponectin accompany resistance to dietary-induced obesity and infertility in female C57BL/6J mice. Int J Obes (Lond). 2007;31:395–402. 16865100 10.1038/sj.ijo.0803392

